# Direct Magnetic Relief Recording Using As_40_S_60_: Mn–Se Nanocomposite Multilayer Structures

**DOI:** 10.1186/s11671-017-2060-6

**Published:** 2017-04-20

**Authors:** A. Stronski, E. Achimova, O. Paiuk, A. Meshalkin, A. Prisacar, G. Triduh, P. Oleksenko, P. Lytvyn

**Affiliations:** 1 V.Lashkaryov Institute of Semiconductor Physics, National Academy of Sciences in Ukraine, 41 Nauki ave, Kiev, 03028 Ukraine; 2 Institute of Applied Physics, Academy of Sciences in Moldova, 5 Academiei str., Chisinau, 2028 Moldova

**Keywords:** Multilayer nanostructures, Diffraction gratings, Magnetic relief, Chalcogenide glasses, Direct recording

## Abstract

Processes of holographic recording of surface relief structures using As_2_S_3_:Mn–Se multilayer nanostructures as registering media were studied in this paper. Optical properties of As_2_S_3_:Mn, Se layers, and As_2_S_3_:Mn–Se multilayer nanostructures were investigated. Values of optical bandgaps were obtained from Tauc dependencies. Surface relief diffraction gratings were recorded. Direct one-stage formation of surface relief using multilayer nanostructures is considered. For the first time, possibility of direct formation of magnetic relief simultaneous with surface relief formation under optical recording using As_2_S_3_:Mn–Se multilayer nanostructures is shown.

## Background

Chalcogenide glasses (ChGs) are typical representatives of non-oxide glasses. ChGs are very promising versatile functional materials for use in optoelectronics as high-speed optical elements, for applications such as data processing devices, electronic switches, and other optical elements. ChGs possess unique characteristics which are different from other glasses: photoinduced phenomena, broad optical transmission window, high linear refractive index (*n* ≈ 2–3), and high optical non-linearity (around two orders of magnitude higher than silica, this makes them suitable for ultra-fast switching in telecommunication systems). These materials and their properties were reviewed in a number of books and review papers [1–6]. ChGs transmit to longer wavelengths in the IR than silica and fluoride glasses. ChGs based on sulfur, selenium, and tellurium typically transmit up to around 10, 15, and 20 μm, respectively [5]. In spite of a wide range of compositions in binary, ternary, and more complex systems of chalcogenide glasses, the problem of modification of parameters still exists. Such modifications can be performed partially by the special technologies (cooling rate, thin film deposition, exposure by light, e-beams or ion beams), by modification, or by creating complex artificial structures [7–13].

The properties of ChGs can be changed by doping with transitional metals or rare-earth elements resulting in change of thermal, optical, luminescent, and magnetic properties [14, 15]. Also the properties of ChGs can be changed by external light-, electron-, or ion-beam source resulting in change of refractive index, optical transmittance, volume (thickness), and viscosity [6, 16, 17]. Based on the changes of these parameters, different optical elements (lenses, gratings, beam splitters, waveguides, etc.) on micro/nanoscale can be fabricated by laser/electron- irradiation directly or followed by chemical development [6].

Composite nanomultilayer structures on the base of chalcogenide glasses are particularly interesting because they enable to realize one-step direct recording of surface relief without selective etching [18–28]. Multilayer structures are the simplest artificial nanostructures which can be rather easily produced with controlled geometrical parameters and investigated as thin films. It is essential, since the changes of the optical parameters (blue shift of the fundamental absorption edge, quantum states, luminescence), as well as of the conductivity and melting temperature (stability), are characteristic and usually examined in nanostructures. A lot of efforts were made to find classic quantum effects, to influence the structure, stability, and thermodynamic parameters of the chalcogenide material in very thin layers (see for example a review of Tanaka [16]), but the obtained results up to now are mostly connected to the optical recording. In this paper, results of direct surface and magnetic relief recording using As_2_S_3_:Mn 2 wt.%–Se nanostructures are considered.

## Methods

The As_2_S_3_ glasses with manganese concentration 2 wt.% were prepared by standard melt-quenching technique using constituent elements of 6 N purity in vacuum-sealed silica ampoules. Ampoules were heated at 80 K/h rate, melt was held at 1010 K during 80 h with subsequent quenching in the air at 10 K/h rate.

**Fig. 1:**
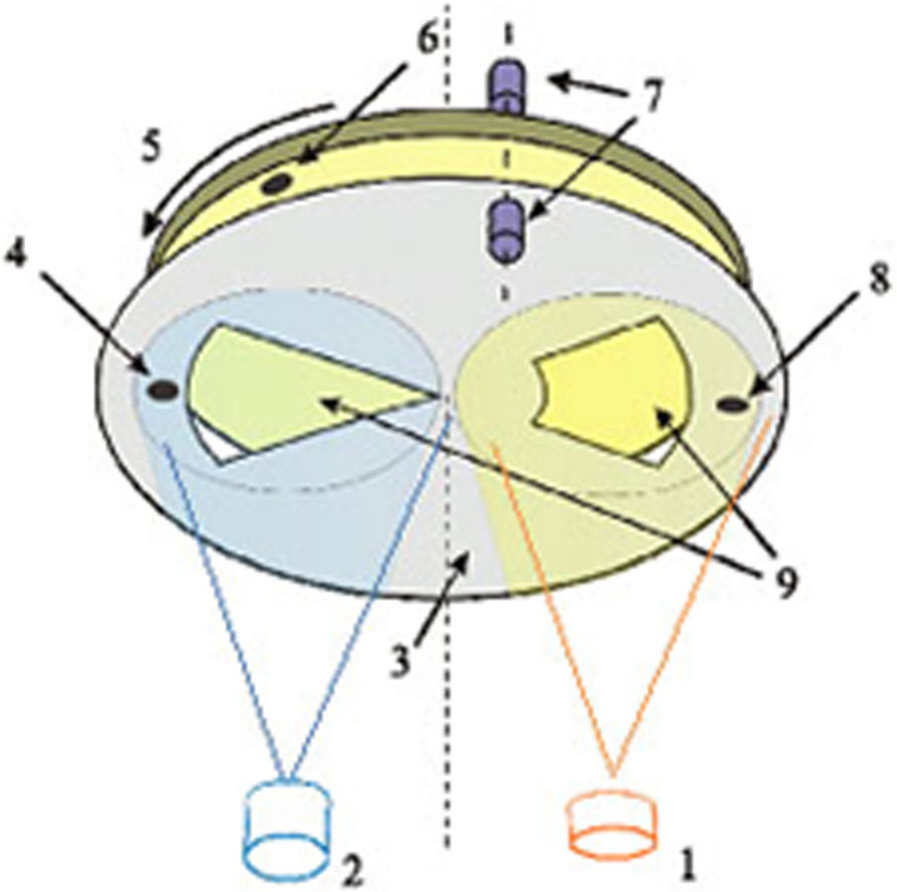
Scheme of device for fabrication of multilayer nanocomposites on the base of chalcogenide glasses. (*1*) As_2_S_3_:Mn evaporator, (*2*) evaporator of Se, (*3*) stationary mask, (*4*), (*8*) quartz thickness sensors fixed on mask, (*5*) rotating sample holder, (*6*) quartz thickness sensor fixed on rotating sample holder, (*7*) optical fibers of the spectrophotometer, (*9*) windows in mask

Composite nanomultilayer structures on the base of chalcogenide glasses were prepared by co-condensation in vacuum. The scheme of the experimental setup is shown in Fig. 1.

**Fig. 2:**
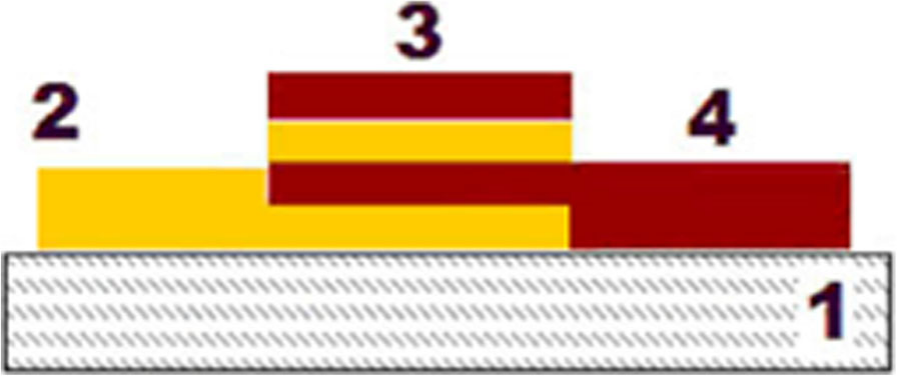
Sample scheme: *1*—glass substrate; *2*—As_2_S_3_:Mn 2 wt.% layer by layer; *3*—As_2_S_3_:Mn 2 wt.%/Se nanostructure; *4*—Se layer by layer

Scheme of samples cross section is shown in Fig. 2.

The overlapping part of samples (Fig. 2) contains alternating nanolayers of Se and As_2_S_3_:Mn 2 wt.%, i.e., two wide rings overlap in the central part of the substrate-forming nanostructure. Outside and internal rings of layers on the substrate contain pure compositions of Se and As_2_S_3_:Mn 2 wt.%, respectively.

We obtained the layers of Se and As_2_S_3_:Mn at the same time on the same substrate consequently through the windows in mask, and they were used to check the composition and calculate the ratio of the layer thicknesses in one modulation period *N* (the total thickness of one Se and As_2_S_3_:Mn nanolayers). Modulation period was ~21 nm = 10 nm + 11 nm; number of periods 90; thickness of one As_2_S_3_:Mn 2% layer *d* ≈ 11 nm; thickness of one Se layer *d* ≈ 10 nm; and total structure thickness ~2000 nm.

The amorphous nature of the samples was verified at room temperature by X-ray diffraction (XRD) technique using a ARL X’tra (Thermo Scientific) diffractometer equipped with a copper tube. The voltage on the tube amounted to 45 kV and current 30 mA. The scattering intensities were measured over an angular range of 2° ≤ *θ* ≤ 140° with a step size of Δ(*θ*) = 0.2° and a count time of 5 s per step.

Obtained films and composite nanomultilayer structures on the base of chalcogenide glasses were investigated using UV–vis spectroscopy. Transmission spectra were obtained in the region 200–900 nm with the use of a spectrophotometer Specord M40.

Morphology of the obtained films and surface relief of the obtained gratings were studied by atomic force microscopy (AFM) with the use of a Nanoscope-IIIa AFM.

Magnetizations of samples were measured with a Cryogenic S600 Super-conducting Quantum Interference Device (SQUID) magnetometer in the temperature range of 3–300 K and in the magnetic field up to 6 T. Measurements of magnetic properties (temperature dependence of the specific magnetic moment) were performed under the different conditions of samples cooling. The sample was cooled in a zero external magnetic field, and then the setting of the magnetic field with specified magnitude was performed. In the following, the magnetic field was remained constant during the sample heating. The interval of temperature change was chosen in such a way that the maximal value of temperature exceeded the temperature of transition into the paramagnetic state. Such dependencies in the following are denoted as ZFC [15]. Further, a sample was cooled in the magnetic field and *M* = *M*(*T*) was obtained, such regime was denoted as FC [15].

Diffraction gratings with 1- and 1.5-μm period were recorded by two laser beams using different light polarization with synchronous diffraction efficiency measurement by red laser (*λ* = 650 nm) in the first diffraction order. Monomode diode-pumped solid state (DPSS) green laser (*λ* = 532 nm) with average spot power density from 150 up to 350 mW/cm^2^ was used.

Local magnetic properties of the surface relief gratings which were fabricated using As_2_S_3_:Mn–Se multilayer nanostructures were investigated using gradient magnetic force microscopy (MFM). MFM measurements were carried out using a scanning probe microscope NanoScope IIIa Dimension 3000 with the use of a two-scan method.

## Results and Discussion

**Fig. 3:**
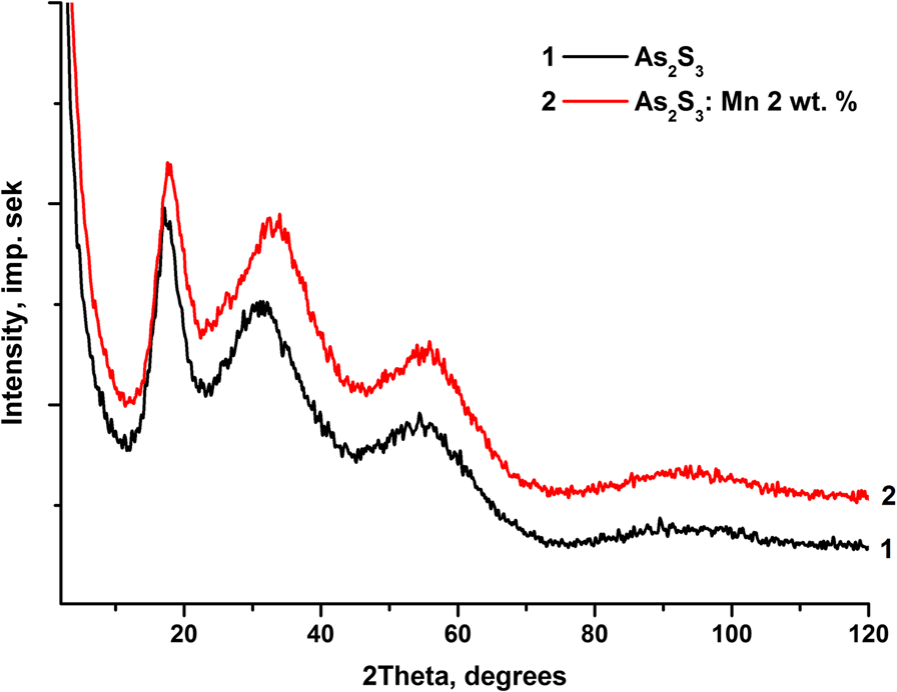
X-ray diffraction patterns for As_2_S_3_ (*1*) and As_2_S_3_:Mn glasses (*2*)

**Fig. 4:**
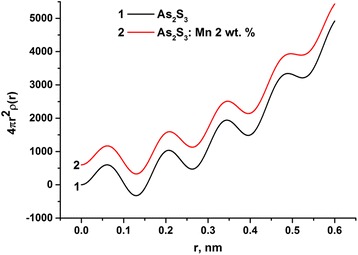
Radial distribution functions for As_2_S_3_ (*1*) and As_2_S_3_:Mn 2% glasses (*2*)

X-ray diffraction patterns confirm the amorphous nature of the bulk samples of chalcogenide glasses (Fig. 3). Spectra are shifted on some distance for better observation. Calculated radial distribution functions have shown no significant change with the introduction of manganese (Fig. 4).

### Magnetic Properties

**Fig. 5:**
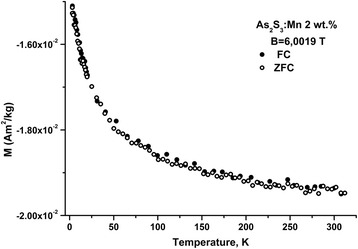
Dependence of mass magnetization *M* on temperature for As_2_S_3_:Mn

ChGs are diamagnetics, in particular As_2_S_3_ glass. Introduction of Mn dopant changes magnetic properties of glasses. Thus, in constant magnetic field dependence of mass magnetization *M* = *M*(*T*) is observed which is characteristic for paramagnetics and ferromagnetics in paramagnetic region of temperature (Fig. 5) and described by Curie–Weiss law.

### Optical Properties

**Fig. 6:**
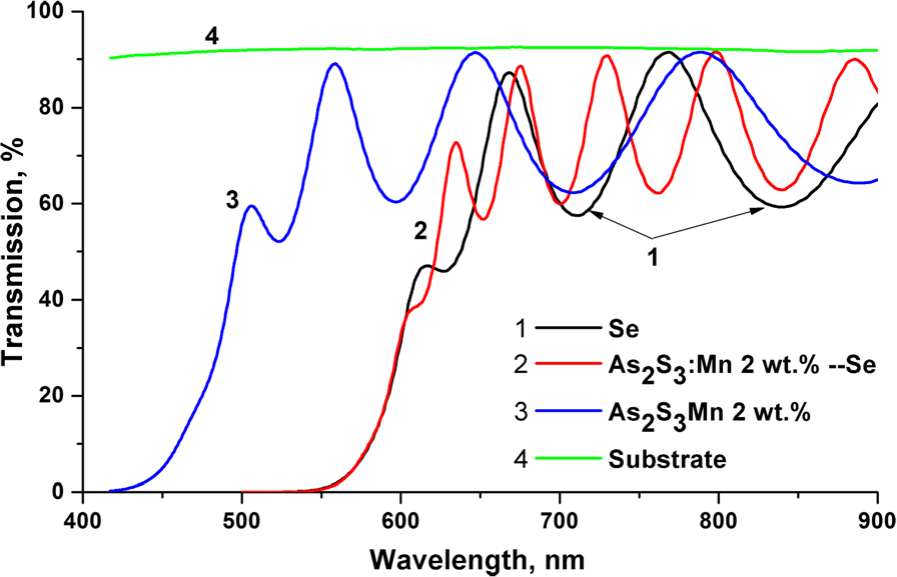
Transmission spectra of As_2_S_3_:Mn 2 wt.% layers, Se layers, and As_2_S_3_:Mn 2 wt.%/Se multilayer structure

Transmission spectra of As_2_S_3_:Mn 2 wt.% layers, Se layers, and As_2_S_3_:Mn 2 wt.%/Se multilayer structures are shown in Fig. 6.

Optical constants of layers were obtained from transmission spectra using Swanepoel method [29] and analyzed within the frames of a single oscillator model. The absorption edge was determined by the relation *α × hν =* const (*hν − E*_g_)^2^, where *hν* is the energy of light quantum, *E*_g_—optical bandgap, and *α*—absorption coefficient. *E*_g_ values were as follows: Se—1.93 eV, As_2_S_3_:Mn 2 wt.%—2.32 eV, composite As_2_S_3_:Mn 2 wt.%/Se layer—1.94 eV. Absorption edge of the multilayer structure As_2_S_3_:Mn 2%/Se is close to the absorption edge of the optical gap of the Se layer (see Fig. 6).

Optical properties were analyzed within the frame of a single oscillator model [29]. According to this model, refractive index *n* is related to energy of incident photon *E* by equation *n* ^2^ *−* 1 *= E*_d_ *E*_0_/*E*_0_^2^ *− E* ^2^ where *E*_0_ is single oscillator energy and *E*_d_ is dispersion energy.

In this expression *E*_0_ determines the position of the effective oscillator connected with an average energy gap and *E*_d_ is dispersion energy characterizing the strength of interband transitions. *E*_0_ and *E*_d_ values were obtained from plots (*n* ^2^ *−* 1)^*−*1^ *= f*(*E* ^2^) using least squares fitting method to straight line. Parameters of the single oscillator model for As_2_S_3_ Mn 2 wt.% and Se layers, and As_2_S_3_:Mn 2 wt.%/Se nanomultilayer structure were obtained from such plots together with optical bandgap values obtained using Tauc plot *αhν* = const (*hν − E*_g_)^2^ are presented in Table 1. Obtained values of optical bandgaps *E*_g_ (see Table 1) for Se layers (1.93 eV) and As_2_S_3_:Mn 2 wt.%/Se nanomultilayer structures (1.94 eV) are close to each other. From Fig. 6, it can be seen also that absorption edges for Se layers and As_2_S_3_:Mn/Se nanomultilayer structure almost coincide.

**Table 1:** Parameters of the single oscillator model for As_2_S_3_:Mn 2 wt.% and Se layers, and As_2_S_3_:Mn 2 wt.%–Se nanomultilayer structure

Layer composition	*n*(0)	*E*_d_, eV	*E*_0_, eV	*E*_g_, eV
Se	2.284	15.086	3.577	1.93
As_2_S_3_:Mn 2 wt.%	2.248	18.696	4.611	2.32
As_2_S_3_:Mn 2 wt.%–Se	2.286	16.714	3.594	1.94

Here, it is necessary to mention that the doping of chalcogenide glasses by transitional metals and rare-earth element changes besides optical, structural, and magnetic properties also changes thermal and luminescent properties of chalcogenide glasses [15].

### Holographic Grating Recording

**Fig. 7:**
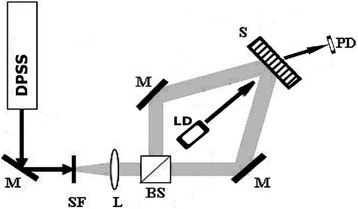
Scheme of diffraction grating recording: *DPSS*—laser; *SF* and *L*—collimator; *BS*—beam splitter; *M*—flat mirrors; *S*—registering media (sample); *LD*—LED; *PD*—registering unit

**Fig. 8:**
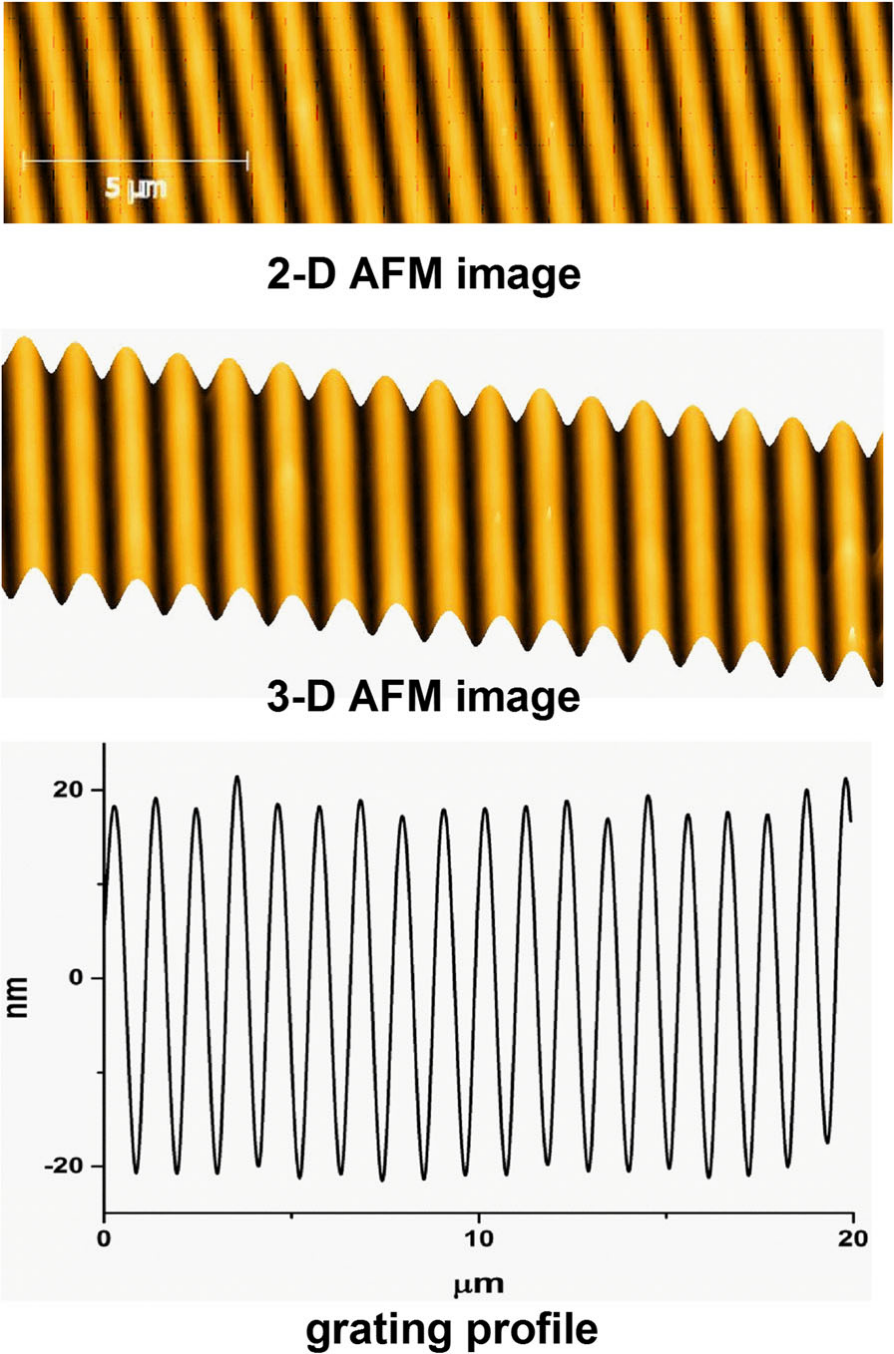
AFM image of recorded holographic grating using As_2_S_3_:Mn–Se nanomultilayers. Spatial frequency ~900 mm^−1^

**Fig. 9:**
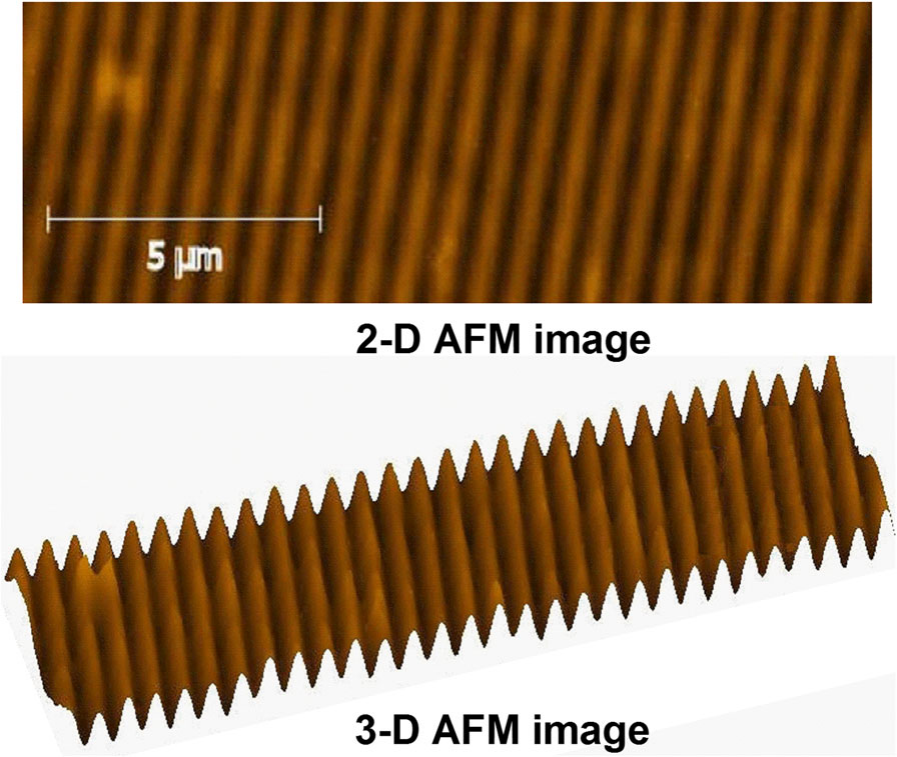
The morphology of surface diffraction gratings recorded on As_2_S_3_:Mn–Se nanomultilayers. Spatial frequency ~1500 mm^−1^, relief height ~26 nm

Scheme of diffraction grating recording is shown in Fig. 7. Gratings were recorded with spatial frequency ~900 mm^−1^; for recording green laser wavelength, 532 nm was used; red laser wave length 650 nm was used for readout. Diffraction efficiency of recorded gratings was ~7% in transmission on 650 nm wavelength, absolute values. AFM image of recorded grating is shown in Fig. 8. It can be seen that obtained relief quality is high. Relief height is ~40 nm. Also gratings with spatial frequency ~1500 mm^−1^ were recorded (Fig. 9).

**Fig. 10:**
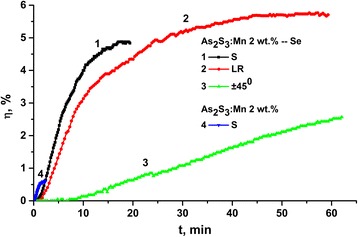
Kinetics of holographic grating recording. As_2_S_3_:Mn 2 wt.%–Se nanomultilayer structures: *1*—S polarization, *2*—circular polarization, *3*—±45° polarization. As_2_S_3_:Mn 2 wt.% layers: *4*—S polarization

Kinetics of grating recording has shown dependence on laser beam polarization. During grating recording in such media (Fig. 10), recording process includes photostimulated changes of refractive index, volume expansion, and mass transfer. The process of grating recording using As_2_S_3_:Mn 2 wt.% layers begins right after switching on the laser illumination. A rapid increase of diffraction efficiency *η* of the grating is observed at the beginning of the recording process (Fig. 10, curve 4). After reaching the maximum, a *η* decrease of gratings follows.

The light polarizations in this experiment were to the grating vector (S polarization), the left–right circular polarization (L–R), and the linear polarization at +45° direction in one writing beam and –45° in the other one. Under the holographic recording using the two linear (S–S) polarized beams or L–R polarizations, we observed at initial stage of exposure linear dependence of *η* growth on time of exposure with a further stage leading to saturation (Fig. 9). The two linear polarized beams falling on the sample surface under angles of polarization (+45° to −45°) produce the linear dependence of *η* growth according to our measurements. Dependence of holographic grating recording on polarization of recording beams when using nanomultilayer structures on the base of chalcogenide glasses was also observed for other compositions of nanomultilayer structures [25, 27].

Interdiffusion and stress relaxation, combined with other photostimulated processes, may be involved in such expansion effects in nanomultilayer structures. The physical base of the process is considered interdiffusion of α-Se and As_2_S_3_ resulting in total volume increase in comparison with total volume of separated sublayers, and the effective intermixing of components at short, nanometer size distances. Competition between stress-induced atomic flux (towards irradiated regions of the film) and the diffusion flux induced by an increase in the bulk energy due to broken bonds (and directed from irradiated to dark regions) can result in either positive or negative net mass transfer in the irradiated region [30–34].Taking into account the change of magnetic properties of As_2_S_3_ glass after introduction of Mn, abovementioned interdiffusion and intermixing of layers and lateral diffusion in nanomultilayer structures, we expected that by using As_2_S_3_:Mn/Se nanomultilayer structures it would be possible to obtain magnetic relief (besides the surface one) during holographic grating recording.

### Magnetic Relief Formation

**Fig. 11:**
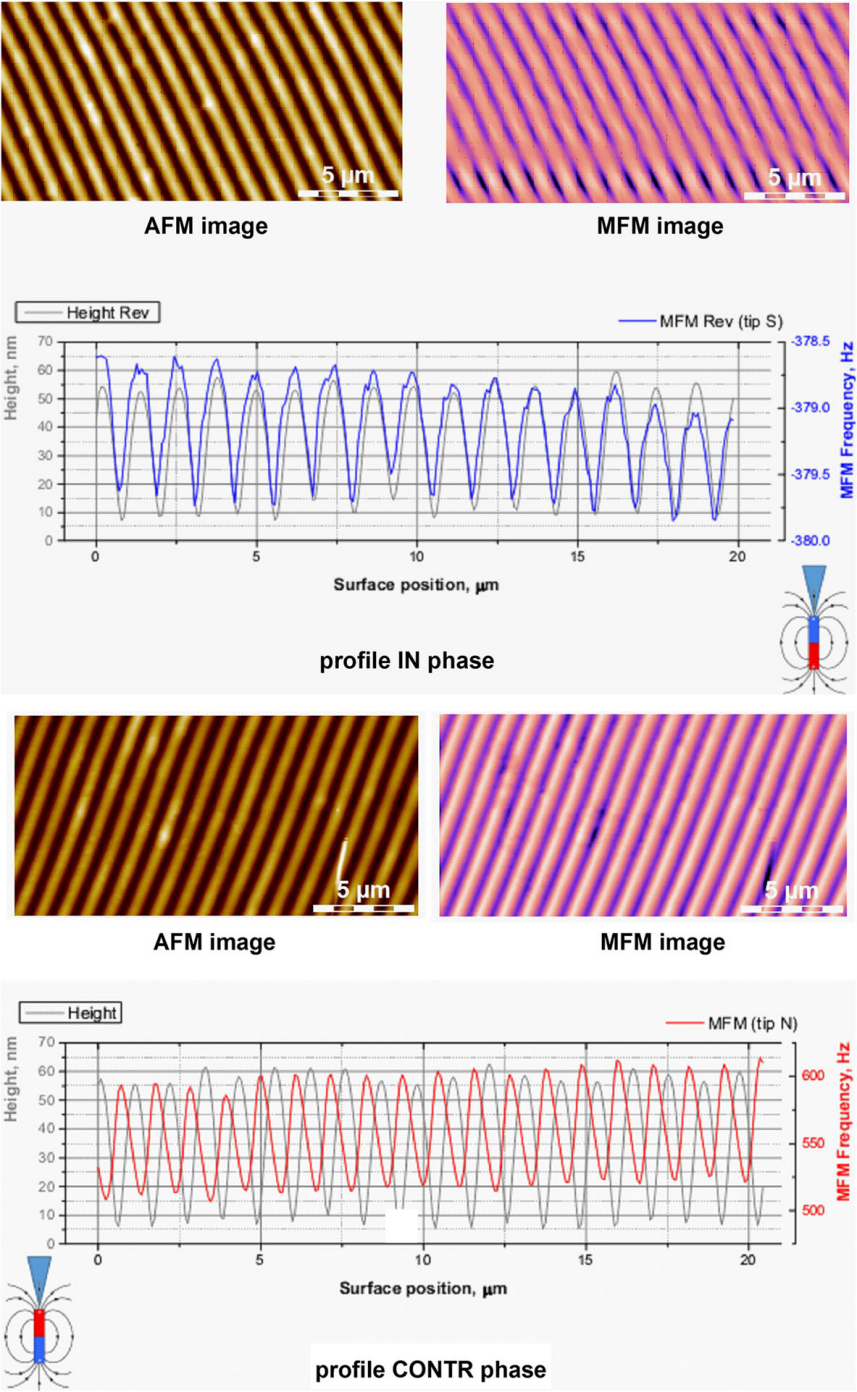
AFM image of grating surface relief and MFM image of magnetic relief for different directions of tip magnetization and also surface relief and magnetic relief profiles

Local magnetic properties of grating surface relief were studied using gradient magnetic force microscopy. AFM and MFM images of the recorded reliefs are shown in Fig. 11.

MFM images show that distribution and value of magnetic field correlates with grating relief in phase or counter phase depending on the tip magnetization direction. Possibility of direct one-step magnetic relief formation during grating recording using As_40_S_60_:Mn 2 wt.%/Se nanomultilayer structures (or similar ones) can be used for creation of surface relief optical elements with unique properties and in magnetic memory applications.

## Conclusions

The presented results demonstrated a direct, one-step process of holographic recording by green light beam of surface relief structures using As_2_S_3_:Mn 2 wt.%/Se nanomultilayer structures.

Due to the changes in transmission, reflection, and in thickness under the influence of laser irradiation, As_40_S_60_:Mn 2 wt.%/Se nanomultilayer structures may be used for effective amplitude-phase optical information recording, for the production of surface relief optical elements with unique properties.

For the first time, it was shown that direct one-step magnetic relief formation is possible during grating recording using As_40_S_60_:Mn 2 wt.%/Se nanomultilayer structures.
